# The natural history and genetic diversity of *Haemophilus influenzae* infecting the airways of adults with cystic fibrosis

**DOI:** 10.1038/s41598-022-19240-2

**Published:** 2022-09-21

**Authors:** Conrad Izydorczyk, Barbara J. Waddell, Robert B. Weyant, Michael G. Surette, Ranjani Somayaji, Harvey R. Rabin, John M. Conly, Deirdre L. Church, Michael D. Parkins

**Affiliations:** 1 Department of Microbiology, Immunology and Infectious Diseases, Cumming School of Medicine, Calgary Adult Cystic Fibrosis Clinic, University of Calgary, Calgary, AB Canada; 2 Department of Medicine, University of Alberta, Edmonton, AB Canada; 3 Department of Medicine, Cumming School of Medicine, Alberta Health Services, University of Calgary, Calgary, AB Canada; 4 Department of Biochemistry and Biomedical Sciences, McMaster University, Hamilton, ON Canada; 5 Snyder Institute for Chronic Diseases, Cumming School of Medicine, Alberta Health Services, University of Calgary, Calgary, AB Canada; 6 Department of Pathology and Laboratory Medicine, Cumming School of Medicine, Alberta Health Services, University of Calgary, Calgary, AB Canada

**Keywords:** Microbiology, Microbial genetics

## Abstract

*Haemophilus influenzae* is a Gram-negative pathobiont, frequently recovered from the airways of persons with cystic fibrosis (pwCF). Previous studies of *H. influenzae* infection dynamics and transmission in CF predominantly used molecular methods, lacking resolution. In this retrospective cohort study, representative yearly *H. influenzae* isolates from all pwCF attending the Calgary Adult CF Clinic with *H. influenzae* positive sputum cultures between 2002 and 2016 were typed by pulsed-field gel electrophoresis. Isolates with shared pulsotypes common to ≥ 2 pwCF were sequenced by Illumina MiSeq. Phylogenetic and pangenomic analyses were used to assess genetic relatedness within shared pulsotypes, and epidemiological investigations were performed to assess potential for healthcare associated transmission. *H. influenzae* infection was observed to be common (33% of patients followed) and dynamic in pwCF. Most infected pwCF exhibited serial infections with new pulsotypes (75% of pwCF with ≥ 2 positive cultures), with up to four distinct pulsotypes identified from individual patients. Prolonged infection by a single pulsotype was only rarely observed. Intra-patient genetic diversity was observed at the single-nucleotide polymorphism and gene content levels. Seven shared pulsotypes encompassing 39% of pwCF with *H. influenzae* infection were identified, but there was no evidence, within our sampling scheme, of direct patient-to-patient infection transmission.

## Introduction

*Haemophilus influenzae* is a Gram-negative pathobiont frequently colonizing the upper respiratory tracts of healthy and chronically ill individuals alike^1,2^. It is among the early colonizers of the cystic fibrosis (CF) lung environment^3^, with most infections caused by non-typeable *H. influenzae* (NTHi) strains^4–6^. While conflicting evidence exists regarding the extent to which *H. influenzae* infection adversely impacts persons with CF (pwCF), the potential for harm is highlighted by the facts that the lower airways are not typically colonized in healthy individuals and that carriage of *H. influenzae* in the lower airways in CF has been associated with increased inflammatory markers^7,8^. Furthermore, *H. influenzae* has the potential to form biofilms in the CF lung^9^, which is associated with disease when formed by other organisms^10–13^.

A limited number of studies have previously observed *H. influenzae* infection to be a dynamic process in CF^4–6,14^. An observation common to these works is that strain replacement over time is frequent and prolonged infection by an individual strain is rare. However, a limitation of these studies is that strains were typically defined only with single molecular methods (i.e. random amplified polymorphic DNA polymerase chain reaction (RAPD-PCR) or pulsed-field gel electrophoresis (PFGE)), restricting their resolution^4,5,14^. This latter point is particularly important, as these studies also frequently observed a small proportion (~ 4.3–34.5%) of shared strains (common to ≥ 2 patients)^4–6,14^, which were interpreted as evidence of possible patient-to-patient transmission. Indeed, shared strains have frequently been used to infer instances of transmission in CF, alongside supporting epidemiological data^13,15–18^. However, it has recently been suggested that even whole-genome sequence (WGS) typing schemes that compare genomes on only core genome single nucleotide polymorphisms (SNPs) (that is, only SNPs in genes present among all isolates compared) are not sufficient to accurately infer transmission between isolates of the same multi-locus sequence typing (MLST) sequence type (ST)^19^, reinforcing that molecular approaches in isolation are similarly inadequate. Only a single study^6^ used whole-genome sequencing (WGS) to analyze 24 *H. influenzae* isolates, but only seven patients had ≥ 2 isolates sequenced, and the study spanned only one year. Previous studies were similarly limited by their time frames (2–7 years), included limited numbers of predominantly pediatric patients, and examined few (sequential) isolates^4,5,14^.

The goal of this study was to investigate the natural history, genomic relatedness, and potential for patient-to-patient cross-infection of *H. influenzae* among adults with CF attending a single North American clinic. We utilized PFGE and WGS in concert to assess *H. influenzae* relatedness in one of the largest adult CF cohorts to date, spanning fourteen years—and at a greater resolution than previous works^5^. We hypothesized that shared strains would be observed in a minority of patients, but that transmission between patients was not a source of new infections.

## Results

### Patient and sample population

**Figure 1:**
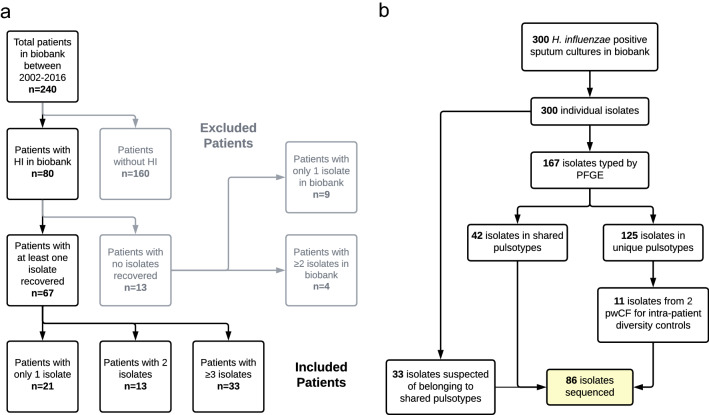
Flowcharts detailing (**a**) the breakdown of the number of included and excluded pwCF in the study and (**b**) the number of isolates at various stages of the study. In (**a**), the black and grey boxes/arrows/text indicate included and excluded patients, respectively. In (**b**), the yellow box indicates the endpoint of isolate selection for sequencing. The total number of isolates typed from the 67 included patients (black box 3 in (**a**)) is 167 (box 3 in (**b**)).

Eighty of 240 non-transplanted pwCF (33.3%) attending the Southern Alberta Adult CF Clinic (also known as the Calgary Adult CF Clinic) between 2002 and 2016 had ≥ 1 *H. influenzae* positive sputum cultures. Patient characteristics are detailed in Table 1. Patients had a median of two positive sputum cultures (range 1–27), with 37.5% having a single positive culture. In total, 300 *H. influenzae* sputum cultures, corresponding to 300 *H. influenzae* isolates, were identified from the biobank. At no time was more than one morphotype of *H. influenzae* identified from any individual sputum culture. Isolates from 13/80 patients (16.25%) were not recoverable or did not grow from preserved samples, but these individuals did not differ by age, sex, dF508 homozygosity, pancreatic status, or FEV_1_% at first isolate from those with recovered isolates (*p* > 0.05). These thirteen patients were more likely to have only a single *H. influenzae* isolate in the biobank (9/13 patients vs. 21/67 with typed isolates) (Fisher’s Exact Test *p* = 0.01) (Fig. 1). From the 67 individuals where biobanked isolates were recovered, 53.7% (n = 36) had ≥ 2 isolates.

**Table 1:** Summary characteristics of patients with at least one isolate typed by PFGE.

Demographics		
Age at first isolation in cohort (median, IQR) (years)	23.02 (19.9–30.54)	
Age at last sample (median, IQR) (years)	27.64 (22.14–36.62)	
Sex (% female)	53.73	
CF Co-morbidities*		
F508del/F508 del (%)	43.94	
F508del/other (%)	36.36	
Pancreatic insufficient (%)	75.76	
Chronic *P. aeruginosa* (%)**	42.62	
Chronic *S. aureus* (%)**	63.93	
CF-related diabetes (%)	9.09	
CF-related liver disease (%)	15.15	
Disease markers*		
FEV_1_ percent predicted (median, IQR)	69.5 (50–87)	
FVC percent predicted (median, IQR)	89.5 (75–105)	
Chronic supplemental oxygen requirements (%)	6.06	
Enteral feeding receipt (%)	7.58	

	At first isolate	Any time
**Chronic medications**		
Inhaled Tobramycin (%)	7.58	15.15
Azithromycin (%)	7.58	15.15
TMP-SMX (%)	4.55	7.81
Inhaled DNase (%)	30.3	50
Inhaled HT Saline (%)	19.4	29.85

*Demographics at entrance into cohort.

**Chronic at any time.

**Figure 2:**
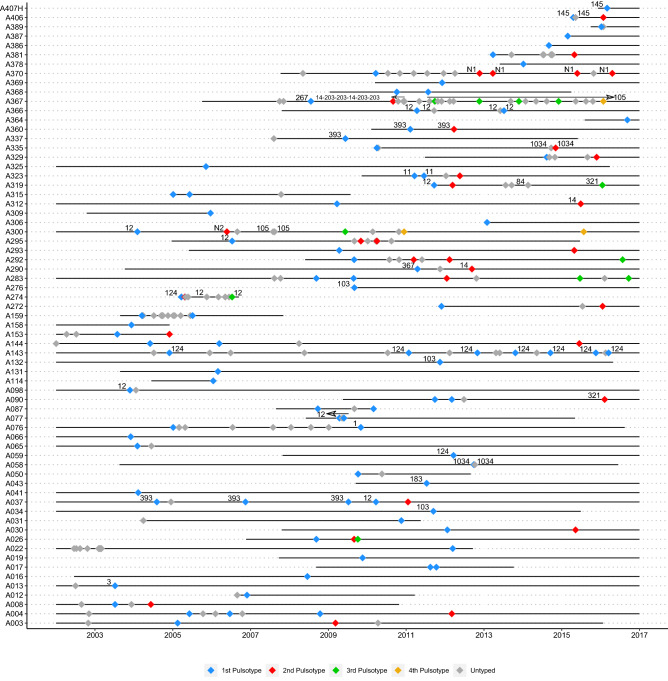
Timeline of pwCF with *H. influenzae* sputum isolated typed by PFGE. pwCF are represented on the Y-axis by patient ID (A###) and the X-axis represents study duration. Each diamond corresponds to a *H. influenzae* positive sputum culture. Colored diamonds represent cultures typed by PFGE, while grey diamonds represent untyped cultures. Colors correspond to sequential pulsotypes in each individual pwCF but have no meaning between pwCF. In most cases, untyped isolates represented serial isolates from patients in whom typed isolates provided a good indicator of strain diversity. STs of sequenced isolates (cultures) are labelled in black. Arrows indicate sequences of cultures belonging to indicated STs.

Focusing on viable first, last, and intermediate yearly isolates when available/when PFGE indicated different pulsotypes among first and last isolates, a total of 136/300 isolates (45.3%) representing at least one isolate from every *H. influenzae* airways culture positive infected individual was typed by PFGE (median two isolates/patient, range 1–7) (Fig. 2). All but two isolates (patient A274, isolate H191 and H192) were non-typeable by *bexB* PCR; in silico serotyping confirmed these as serotype f and belonging to a known serotype f lineage—ST-124^20^. All other ST-124 isolates were either missing genes in the serotype f backbone or had no capsular genes detected.

### Short-term carriage and rapid turnover of *H. influenzae* is commonplace in pwCF

The dynamics of infecting PFGE pulsotype strains are presented in Fig. 2. A median of one pulsotype (range 1–4) was recovered per patient, but the majority (75%) of patients with ≥ 2 isolates typed had ≥ 2 pulsotypes detected over time. Untyped isolates represented serial isolates likely representing the same pulsotype as typed isolates in most cases. The time between different pulsotypes ranged from months to years, and recovery of a previous pulsotype after a second pulsotype was detected was observed only in a single instance. In patient A367, STs 14 and 203 overlapped by a single culture before ST-203 became the new dominant strain. Prolonged infection by individual pulsotypes was also observed in a minority of patients, with durations of up to ten years in one patient. Most patients carried pulsotypes unique to themselves, but seven shared pulsotypes consisting of a median 4 isolates/pulsotype from 26/67 patients (median 3/pulsotype) were found (Supplementary Fig. S1 and Supplementary Table S4).

Isolates belonging to shared pulsotypes (n = 42) underwent WGS, along with 33 isolates suspected of belonging to shared pulsotypes based on collection dates (Fig. 1). Eleven further isolates from two patients with different infection histories (A367, n = 7, multiple unique and shared strains; A370, n = 4, two unique strains) were sequenced as a control to assess intra-patient genetic diversity over different time. Patient A367 was selected as a control because they had the densest sampling of any patient, allowing for the determination of pairwise SNP distances and differences in gene content on a very short timescale. Further, they had two strains—one unique and another ultimately found to be shared (ST-105), indicating a complex infection history. Patient A370, in contrast, was selected because they had yearly isolates available, allowing for the determination of intra-patient SNP distances and differences in gene content over a longer timescale than patient A367. Most of patient A370’s isolates also belonged to a single pulsotype (with the exception of a single isolate collected years prior)—a simpler infection history and valuable contrast to patient A367. In total, 86 isolates were sequenced. The median sequencing depth was 78.5 × and the average assembly length was ~ 1.81Mbp (range ~ 1.66–1.92). The median number of coding sequences annotated per genome was 1784.5 (IQR 1741.75–1866.5).

In silico MLST showed that the seven shared pulsotypes corresponded to thirteen STs (Supplementary Tables S3 and S4). In some cases, this was due to single- or double-locus variants of a dominant ST within a pulsotype, but division into unrelated STs was also observed. Five further STs were identified among additionally sequenced isolates, including two new shared STs not identified by PFGE (ST-11 and ST-105). Infection by shared STs was not associated with patient demographic factors (age, sex, deltaF508 status, pancreatic sufficiency, *p* > 0.05). Due to the unbiased nature of MLST, subsequent analyses were carried out on an ST-specific basis. Recovered STs were a random sample of the broader *H. influenzae* ST pool.

### Intra-ST genetic diversity is prevalent in pwCF

**Figure 3:**
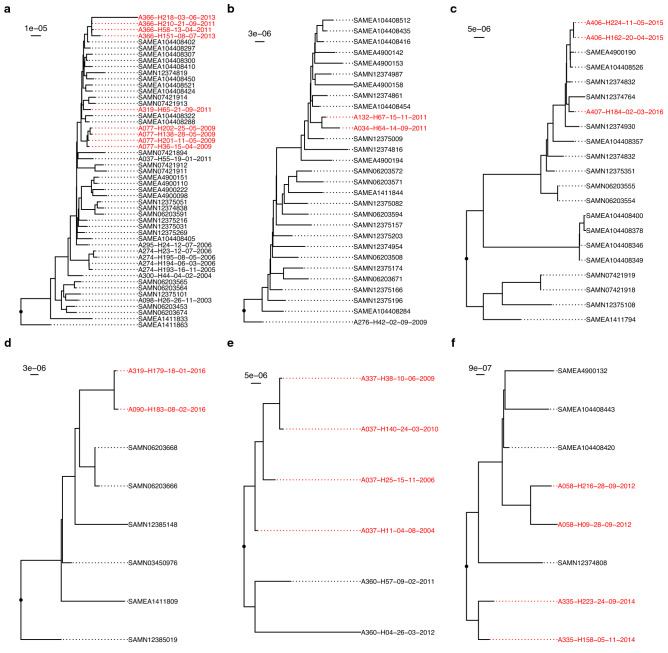
Maximum likelihood consensus phylogenies for STs with patient-pairs of interest. (**a**) ST-12, (**b**) ST-103, (**c**) ST-145, (**d**) ST-321, (**e**) ST-393, and (**f**) ST-1034. Publicly available genomes are indicated by their BioSample accessions. Isolates colored red belong to patient-pairs with possibly transmitted isolates and are separated by p-distances smaller than the threshold (1.68 × 10^−5^ SNPs per site) for potential transmission. Isolate names are given in the format “Patient Number”-“Isolate Number”-“Collection Date (DD-MM-YYYY)”. Scale bars for each tree are given in the units SNPs per site. Branch nodes with > 95% Ultrafast bootstrap support are labelled.

ST-specific SNP phylogenies supplemented with public genomes are presented in Fig. 3 and Supplementary Fig. S2. Where multiple isolates/patient were available, intra-patient isolates always clustered together and displayed clonal relationships. These were typically supported by small SNP p-distances (order of 10^−6^ SNPs/site) (Supplementary Table S5). However, large p-distances (10^−4^ SNPs/site) were also observed in both clonal and non-clonal contexts and over short timeframes. For example, in patients A367 and A143 (Supplementary Fig. S2), large distances were associated with intra-patient sub-clades and non-chronological ordering of isolates. In contrast, patient A370’s ST-Novel 1 isolates appeared clonal in their phylogeny but were separated by distances as large as 10^−4^ SNPs/site (Supplementary Fig. S2). Interspersal of CF isolates with public genomes indicated patient-specific isolates were a random sample from the broader pool of diversity within a given ST.

**Figure 4:**
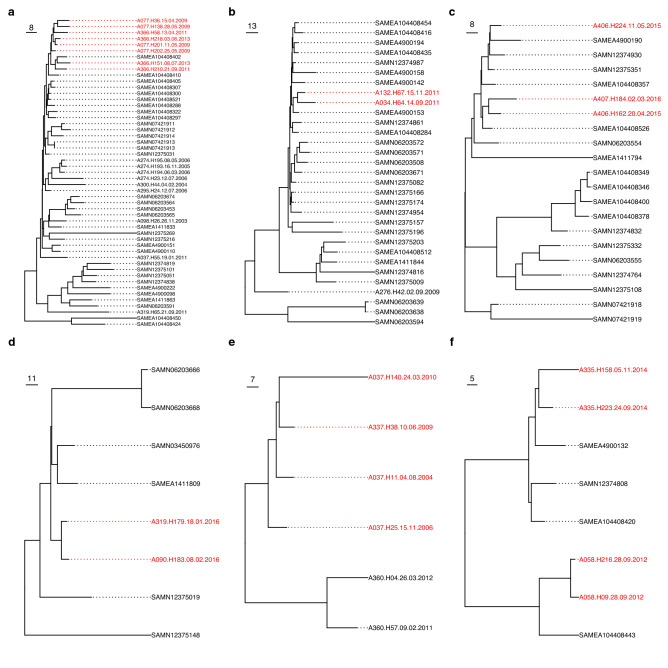
Neighbor-joining trees for STs with patient-pairs of interest generated using differences in gene content. (**a**) ST-12, (**b**) ST-103, (**c**) ST-145, (**d**) ST-321, (**e**) ST-393, and (**f**) ST-1034. Isolates colored red belong to patient-pairs of interest. Public genomes are labelled with their BioSample accessions. Isolate names are given in the format “Patient Number”-“Isolate Number”-“Collection Date (DD-MM-YYYY)”. Scale bars for each tree are given in number of differences in gene content.

Intra-patient isolates differed by a median of 24 genes (IQR 16.5–38.5) and clustered by patient based on accessory gene content in most instances (Fig. 4 and Supplementary Fig. S3). Inter-patient differences in gene content were significantly larger (median 50 genes, Mann Whitney U-test *p* < 0.001) but overlap of these distributions was observed (Supplementary Fig. S4 and Supplementary Table S6). This was primarily driven by small inter-patient differences in gene content among ST-12 isolates. In a few instances, intra-patient diversity was evident in the separate clustering of isolates (patient A406, ST0145) and localization of isolates on relatively long branches (A274, ST-12). Five instances of multi-patient clusters were observed (see below).

### Putative hypermutators are common amongst pwCF

Of the 76 isolates involved in ST-specific SNP calling (isolates not belonging to a shared ST were not tested), eighteen (23.7%) displayed elevated Ts/Tv ratios (> 3) and/or had mutations in genes involved in hypermutation (Supplementary Table S7). Isolates with both elevated Ts/Tv ratios and mutations were observed in patients A367 (ST-105, 6 isolates), A366 (ST-12, 1 isolate), and A370 (ST-Novel 1, 4 isolates). Among these isolates, missense/frameshift mutations were observed in *mutL*, *mutM*, *mutS*, and *mutT*, and multiple mutations/isolate were observed among some of patient A367’s and A370’s isolates, including at adjacent amino acid positions. Isolates with only elevated Ts/Tv ratios were observed in one patient in each of STs 105 (A367, 1 isolate), 203 (A367, 4 isolates) and 393 (A360, 2 isolates). Another isolate from patient A367 (ST-105, isolate A367-H209) had a frameshift mutation with predicted loss of function in *mutT* and a missense mutation in *mutS*, both of which appear in later isolates, but did not display an elevated Ts/Tv ratio. Accordingly, this isolate may have been in the early stages of hypermutation and the source of these mutations in later isolates. Only patient A367 had putative hypermutators belonging to two different STs (STs 105 and 203), and two patients (A077 and A367) had both putative hypermutators and non-hypermutating isolates. Isolates suspected of being hypermutators had higher pairwise SNP p-distances to other isolates from a patient than non-hypermutating isolates (Supplementary Table S5).

### *H. influenzae* infection transmission is not observed amongst pwCF despite genomic relatedness

A two-tiered approach was used to identify potential infection transmission events. For the first tier, a genetic distance threshold of 1.68 × 10^−5^ SNPs per site was calculated (Supplementary Fig. S5). Twenty-three isolate pairs from eight patient-pairs (median 2 isolate pairs per patient-pair, range 1–8) from six MLST STs passed the first tier and were analyzed simultaneously using the second tier (comprising the four analyses described above).

Phylogenetic support was observed in three patient-pairs (Fig. 3). Two of these pairs (ST-103 and ST-321) had only one isolate/patient available, and phylogenetic support came from the clustering of inter-patient isolates to the exclusion of public genomes. In the third case (ST-393), co-clustering of inter-patient isolates was observed. Isolates clustered by patient, fell on long branches, or inter-patient isolates were interspersed by public genomes for the remaining patient-pairs, indicating a lack of phylogenetic support. Similarly, pangenome support based on gene presence/absence clustering was observed for 5 patient pairs (Fig. 4). Again, in three pairs (A077-A366 (ST-12), A406-A407 (ST-145), and A037-A337 (ST-393)), support was by mixed clustering of inter-patient isolates, to the exclusion of public genomes. Clustering to the exclusion of public genomes alone provided support for pairs A034-A132 (ST-103) and A090-A319 (ST-321), and non-supported pairs clustered by patient or among public genomes.

**Figure 5:**
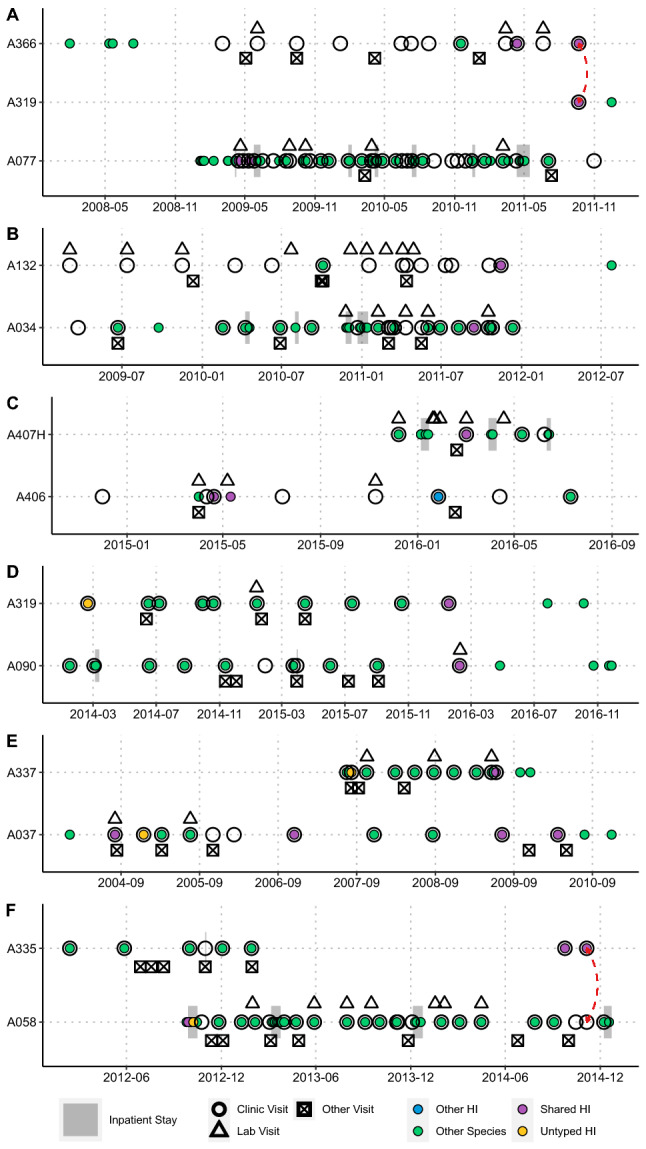
Epidemiological encounter timeline plots—potential for infection transmission events. (**A**) ST-12, (**B**) ST-103, (**C**) ST-145, (**D**) ST-321, (**E**) ST-393, (**F**) ST-1034. pwCF identifiers are given as “A###” on the y-axis. Dates are represented on the x-axis. Sputum cultures are represented by colored circles; color of circle denotes whether that culture contained a shared HI strain (purple), a different HI strain (blue), an untyped HI strain (yellow), or no HI (either culture negative or any other CF pathogen (green)). Clinical encounters are represented by empty black shapes; a circle represents a clinic visit, a triangle represents a lab visit, and an X’d square represents a different type of visit. Grey bars represent in-patient stays. Red arrows indicate dates when involved patients had the same type of clinical encounter on the same day.

Figure 5 depicts all clinical visits of all patient-pairs, divided by ST. Only two instances of possible patient encounters on the same day were observed (patients A366/A319 and A058/A335, Fig. 5A,F). In the latter case, the same-day clinic visit occurred years after the shared ST-1034 isolates were first detected in patient A058. This patient was then *H. influenzae*-free in the years leading up to this same-day clinic visit, making transmission highly unlikely. In the former case, the same-day clinic visit corresponded to detection of the shared ST-12 isolates in both patients. However, both patients had *Staphylococcus aureus* with different antibiograms isolated on this day, suggesting a mix up of culture labelling unlikely. Both patients also entered the adult clinic with histories of significant bacterial load in the airways, and this was patient A319’s first visit; it would be impossible to detect *H. influenzae* at the high bacterial load (10^6^ CFU/ml) that was observed if transmission had occurred on that day. There were no same-day encounters among all other patient-pairs, making transmission unlikely. Table 2 summarizes the results of the transmission analysis. As no patient-pairs were supported by all four analyses, clinic-associated transmission was considered unlikely based on clinic interactions in all cases despite close genetic relatedness among some isolate pairs. We cannot rule out if some type of social interactions may have occurred outside of the healthcare setting.

**Table 2:** Support for infection transmission between patient pairs with potentially transmitted isolates.

Patient pair	ST	Isolate pairs^a^	Distance threshold support	Phylogenetic support	Pangenome support	Carriage overlap^b^	Epidemiological support
A077, A319	12	A077-H36, A319-H65	Yes	No	No	No	No
		A077-H201, A319-H65					
A077, A366	12	A077-H36, A366-H58	Yes	No	Yes	No	No
		A077-H36, A366-H201					
		A077-H138, A366-H58					
		A077-H138, A366-H201					
		A077-H201, A366-H58					
		A077-H201, A366-H201					
		A077-H202, A366-H58					
		A077-H202, A366-H201					
A319, A366	12	A319-H65, A366-H58	Yes	No	No	Yes	Yes
		A319-H65, A366-H210					
A034, A132	103	A034-H64, A132-H67	Yes	Yes	Yes	Yes	No
A406, A407	145	A406-H162, A407-H184	Yes	No	Partial^c^	No	No
		A406-H224, A407-H184					
A090, A321	321	A090-H183, A319-H179	Yes	Yes	Yes	Yes	No
A037, A337	393	A037-H11, A337-H38	Yes	Yes	Yes	No	No
		A037-H25, A337-H38					
		A037-H140, A337-H38					
A058, A335	1034	A058-H09, A335-H158	Yes	No	No	No	No
		A058-H09, A335-H223					
		A058-H216, A335-H158					
		A058-H216, A335-H223					

All isolate pairs that had pairwise p-distances below a threshold of 1.68 × 10^−5^ SNPs per site are included.

^a^Isolate pairs are presented in the format “Patient Number-Isolate Number”.

^b^Carriage overlap refers to detection of the associated *H. influenzae* isolates in both patients within 6 months of each other. It is a sub-category of Epidemiological Support.

^c^Patient A407’s isolate clustered with one of patient A406’s isolates; the second isolate from patient A406 clustered separately from their first.

## Discussion

We analyzed a large *H. influenzae* WGS dataset from pwCF collected over fourteen years. Infection with *H. influenzae* was observed in a similar proportion of our adult CF cohort as noted in previous pediatric studies^4,5^. The infection process itself was dynamic at both the strain and sub-strain levels. Novel strain acquisition and replacement was common, but infection by distinct, overlapping strains was observed in only a single instance. Only a minority of patients carried the same strain for prolonged periods of time. Intra-patient genetic diversity was observed at the SNP and gene content levels, and greater SNP diversity was associated with hypermutation. Consistent with our hypothesis, shared strains and closely related isolates were observed among some patients, but clinic-associated transmission was considered unlikely in all cases based on our combined genomic and epidemiological analyses.

By utilizing the additional resolution offered by WGS in addition to PFGE to type isolates from pwCF, we were able to discriminate between isolates more accurately from different individuals and investigate intra-patient *H. influenzae* infection dynamics at the sub-strain level. Indeed, previous studies of *H. influenzae* in CF used shared pulsotypes as the baseline for inferring possible transmission between patients^4,5^. Here, we showed that neither shared pulsotypes, nor close relatedness at the pan-genome SNP level, were sufficient to indicate direct transmission of *H. influenzae* in pwCF, as has been noted in transmission studies of other pathogens^19^. WGS further allowed us to extend the frequent strain-switching infection dynamics previously observed^4–6,14^ (and observed here as well) to demonstrate that strain diversity over time is not limited to pulsotypes/STs but may exist within strains defined by these methods similarly to other CF pathogens^21^. This diversity, along with the existence of intra-patient sub-clades, was notably observed among potentially hypermutating isolates in this study—similar to findings for *Pseudomonas aeruginosa* in CF^22^. The proportion of potential hypermutators was in agreement with some previous studies^4^ but not in others^5^.

Most pwCF are infected by non-typeable *H. influenzae* (NTHi)^4–6^, and the same was observed here. Only two patients had any isolates with capsular genes present, all of which belonged to serotype f-associated ST-124^20^. Isolates from a third patient with ST-124 isolates were negative for *bexB* and in silico serotyping, indicating a possible, recent loss of the ability to produce/express a capsule among these isolates, as has been reported for some unencapsulated strains^20,23^.

Previous studies of other pathogens in CF have observed that initial infecting strains subsequently expand and diversify in the lungs^24^. Here, we have evidence to support these findings for *H. influenzae* by demonstrating clonal relationships between most intra-patient isolates but also the existence of intra-patient diversity. Coexistence of divergent lineages has been reported with *P. aeruginosa* in CF^21^, and our data also support this as a possibility for *H. influenzae*. Indeed, instances where SNP and/or gene distances were too large between isolates collected too closely in time to represent evolution of a single lineage were also observed^25^, although some could be explained by hypermutation.

Persistent *H. influenzae* isolates recovered from chronic obstructive pulmonary disease (COPD) have previously been observed to not undergo significant gene gain or loss during persistent infection^26^. This contrasts with our findings in pwCF, in which the average difference in gene content was ~ 32 genes. However, as some of our isolates were collected mere days apart, they may represent transient infection by similar but distinct lineages (that is, multiple lineages may be part of the same infection “episode”), rather than persistent colonization (i.e. the detection of the same ST at multiple monthly clinic visits, as defined by the aforementioned study). Additionally, as we utilized draft assemblies, we cannot be sure that at least some of the observed differences were not due to assembly/annotation/ortholog clustering errors, despite significant efforts to control for this.

Our findings of infection by unique strains in most pwCF and a lack of transmission are consistent with previous studies of other pathogens^17,19,27^. Our data suggest that, as for other CF pathogens, most patients likely acquire infection from environmental sources (in the case of *H. influenzae*, primarily non-CF human reservoirs). The proportion of patients with shared strains here is consistent with previous works^4,5^. Our findings of closely related inter-patient isolates that were unlikely to have originated via transmission events due is also supported by similar observations of *H. influenzae* in COPD^25^. Indeed, apart from epidemic strains^18^, infection transmission in CF appears to be a very rare occurrence with infection control standards, with transmission events occurring as isolated events among a background of no transmission between the majority of patients at any given clinic^17,19,27^. Consistent with this is the observation that the shared *H. influenzae* strains identified here appear to be a random sample from the broader *H. influenzae* pool, as previously observed for *P. aeruginosa* and *E. coli* in CF^28,29^. We found no evidence of the circulation of any epidemic strains, as has been reported most notably for *P. aeruginosa* and some members of the *Burkholderia cepacia* complex^18,30^.

The clinical importance of these findings is highlighted primarily in the high frequency of strain switching observed. This data implies a need for consistent monitoring of susceptibility profiles over time, as prolonged infection by a single strain occurs in a minority of patients—contrasting what is increasingly apparent with *P. aeruginosa* from CF^31,32^. Another significant finding is that no likely instances of infection transmission were identified. As the Southern Alberta Adult CF Clinic employs strict infection and prevention control procedures, this data supports the effectiveness of such procedures and their importance in preventing the spread of infections in CF.

We recognize several limitations of this work. Firstly, the retrospective nature of this work means we were limited to a retrospectively collected set of isolates and different isolate collection rates from different patients. Second, because of the magnitude of potential isolates, we used strict strain selection criteria such that not all *H. influenzae* isolates in the biobank were typed by PFGE, nor were all those typed by PFGE sequenced; thus, it is theoretically possible some related isolates thus may have been missed. Indeed, we identified multiple isolates related by MLST that were not identified initially by PFGE. Another limitation is that only one isolate per morphologically distinct colony type was collected from each *H. influenzae* positive sputum culture. This means that any diversity present within a morphologically identical isolates is limited to a single representative isolate; such diversity is increasingly appreciated for *P. aeruginosa* in CF^33^. A single sample per patient per time point further complicates interpretation of phylogenetic relationships, as some relationships typical of transmission cannot be observed. That only isolates from shared STs were sequenced further limits the drawing of broad conclusions from this work, even within the scope of CF. As the epidemiological data available for this work was limited to in- and out-patient clinic visit dates, we were unable to assess the potential for indirect transmission or transmission in community settings. Indeed, unlike *P. aeruginosa* and *B. cepacia* complex, *H. influenzae* and similar pathobionts broadly exist in the general population, who may serve as intermediaries for infection spread, complicating the detection of infection transmission. Lastly, putative hypermutators were not confirmed in the laboratory for elevated mutation rates and some may have been missed, leading to elevated SNP/p-distances and artificially inflated p-distance thresholds.

In summary, we demonstrated that *H. influenzae* infection in pwCF is a complex process distinct from that documented for *P. aeruginosa* and other airway pathogens. *H. influenzae* diversity is present at the strain and sub-strain levels between and within patients. We also confirmed the need for continuing resistance testing of new *H. influenzae* isolates in patients given the high rate of strain switching observed but did not identify any potential instances of healthcare associated infection transmission. Indeed, our data support the conclusion that CF patient-to-patient transmission does not appear to be a significant source of new *H. influenzae* infections, but that closely related strains may be acquired from other sources.

## Methods

### Patient population and strains

In this retrospective longitudinal cohort study, we analyzed *H. influenzae* isolates collected from patients attending the Southern Alberta Adult CF Clinic between January 2002 and December 2016. The clinic provides comprehensive care to all individuals with CF residing in Southern Alberta, Canada, and patients attending the clinic are followed at least quarterly. Serial sputum samples are collected from each individual, (including every outpatient and inpatient encounter) and are analyzed for the presence of CF pathogens^34^. All pathogens identified in real-time from every clinical encounter are frozen at − 80 °C in glycerol and transferred into our biobank. If multiple morphotypes of any pathogen were identified, representative examples of each individual morphotype were included in the biobank. For inclusion, patients had to have a diagnosis of CF, be ≥ 18 years of age and have ≥ 1 *H. influenzae* positive sputum cultures during the time span of the study. Patients were excluded if they had received a double lung transplant or were censored at the time of transplant if they entered the cohort prior to the procedure.

### PFGE, serotyping, DNA extraction, and WGS

In order to efficiently assess for diversity within our large collection, representative isolates from all patients with *H. influenzae* positive sputum cultures between 2002–2016 underwent PFGE using protocols adapted from Parkins et al.^35^. For each patient with at least one positive sputum culture, we aimed to type all viable first, last, and intermediate yearly isolates when present or when PFGE patterns indicated differing pulsotypes between the first and last isolates. Isolates with banding patterns with ≥ 80% similarity and ≤ 3 band differences were considered to represent the same strain^36^. Serotyping of all isolates typed by PFGE was performed by PCR using primers for *bexB* as described by Davis et al.^37^.

DNA from isolates belonging to shared pulsotypes was extracted with the Promega Wizard® Genomic DNA Purification Kit and sequencing libraries prepared using the Nextera XT DNA Library Prep Kit. Where possible, additional isolates collected from the same patients suspected of belonging to shared pulsotypes based on collection dates (including two isolates collected in 2017), as well as longitudinal isolates from two patients to further assess intra-patient diversity, were also included. Isolates belonging to shared pulsotypes were sequenced by the Illumina MiSeq V3 (2 × 300 bp). Sequencing data from two isolates was found to correspond to *H. haemolyticus* (isolate A290-H059) or produced a very poor de novo assembly (isolate A058-H217) and was excluded from all subsequent analyses.

See Supplementary Methods for full details.

### Bioinformatic analyses

Sequencing reads were trimmed using Trimmomatic^38^ (v0.39) and in silico multi-locus sequence typing (MLST) was performed using stringMLST^39^ (v0.6.3). De novo assemblies were generated using Unicycler^40^ (v0.4.8), polished with NextPolish^41^ (v1.3.0), and annotated with RASTtk via the PATRIC CLI^42^ (v1.035). Core and accessory genomes were determined using Panaroo^43^ (v1.2.7), and gene presence/absence clustering was performed with the Ape^44^ (v5.3) package in R.

SNP calling was performed in an MLST sequence type (ST) specific manner with Snippy (v4.6.0) (https://github.com/tseemann/snippy) using a same-ST isolate as a reference (Supplementary Table S1). Phylogenies were generated using IQ-Tree^45^ (v2.0.3) and recombination masked with ClonalFrameML^46^ (v1.12). SNP p-distances were calculated with MEGA X^47^ (v10.2.4).

Transitions/transversion ratios were estimated using VCFTools^48^ (v0.1.6) to identify putative hypermutators. Mutations in hypermutation associated genes were identified with custom Python scripts.

In silico serotyping of all sequenced isolates was additionally performed on those isolates subjected to WGS using the capsule prediction method of Potts et al.^20^.

See Supplementary Methods for full details.

### Transmission analysis

A two-tiered approach was used to assess for transmission between patient-pairs. In the first tier, using the approach of Coll et al.^49^, a genetic distance threshold representing the number of mutations expected to accumulate if isolates of a pair were collected within 6 months of each other and within 6–12 months of the time of their most recent common ancestor (MRCA) was calculated. In the second tier, isolate pairs with a SNP p-distance below this threshold were assessed simultaneously on an ST-basis for fine-scale relatedness using four complementary but independent analyses. These analyses included: (i) phylogenetic support: mixed clustering of isolates from different patients, encompassing of the genetic diversity of one patient within the diversity of another, or clustering between individuals with CF to the exclusion of public genomes (all with ≥ 95% Ultrafast bootstrap support), (ii) pangenome support: mixed clustering of isolates from different patients based on gene presence/absence, (iii) carriage support: detection of the associated *H. influenzae* isolates in both patients within 6 months of each other, and (iv) epidemiological-associations: identification of the potential interaction of patients occupying the same healthcare associated space/time. Epidemiological factors assessed opportunities for patient interaction, including overlapping hospitalizations, clinic visits, and laboratory or radiology facility usage. The cumulative effect of each of these criteria being satisfied would be to support a potential transmission event between patients. Lack of support in any of these analyses was considered an exclusion of possible transmission.

See Supplementary Methods for additional details.

### Public genomes

STs with patient-pairs with potentially transmitted pairs of isolates were supplemented with publicly available genomes from several recent *H. influenzae* WGS studies^20,23,25,26,50,51^ (Supplementary Table S2). These were processed and analyzed as above. See Supplementary Methods for details.

### Statistical analysis

Cohort characteristics were descriptively summarized. Association testing of clinical/demographic factors with included/excluded patients and patients with/without multiple/shared strains was performed in R (v4.1.1) using a Fisher’s Exact Test for count data and a Mann Whitney U-test for comparison of distributions.

### Ethics approval and consent to participate

This study was performed in accordance with the Declaration of Helsinki and was approved by the University of Calgary’s Conjoint Regional Health Ethics Board (REB15-0854 and REB15-2744). Patients were recruited from the Southern Alberta Adult CF Clinic and all patients provided written informed consent for the collection and storage of specimens and subsequent analysis. All samples and patient data were de-identified.

## Supplementary Information

Supplementary Information 1.

Supplementary Information 2.

Supplementary Information 3.

## Data Availability

The datasets generated during and supporting the conclusions of this article are available from the National Center for Biotechnology Information (NCBI) Sequence Read Archive (SRA) repository under the BioProject PRJNA770358 (https://www.ncbi.nlm.nih.gov/bioproject/PRJNA770358). The public datasets supporting the conclusions of this article are similarly available under the BioProject IDs PRJEB23674, PRJEB2400, PRJEB28646, PRJ282520, PRJNA358390, and PRJNA512636. This information is also included within the article and its additional files. High quality Figures and Supplementary Figures, as well as all PFGE gels, are available in an online repository: https://doi.org/10.6084/m9.figshare.c.6044099.v1.
