# Supplementary material for: The natural history and genetic diversity of Haemophilus influenzae infecting the airways of adults with cystic fibrosis
Source: Sci Rep. 2022 Sep 21;12:15765. doi: 10.1038/s41598-022-19240-2 (PMC9492733; doi:10.1038/s41598-022-19240-2)
Supplement: Supplementary file 1 — Supplementary Information 1. [file 41598_2022_19240_MOESM1_ESM.pdf]

Supplementary Figures

Please note that digital versions of all Supplementary Figures are also available from an online repository to facilitate viewing: https://doi.org/10.6084/m9.figshare.c.6044099.v1.

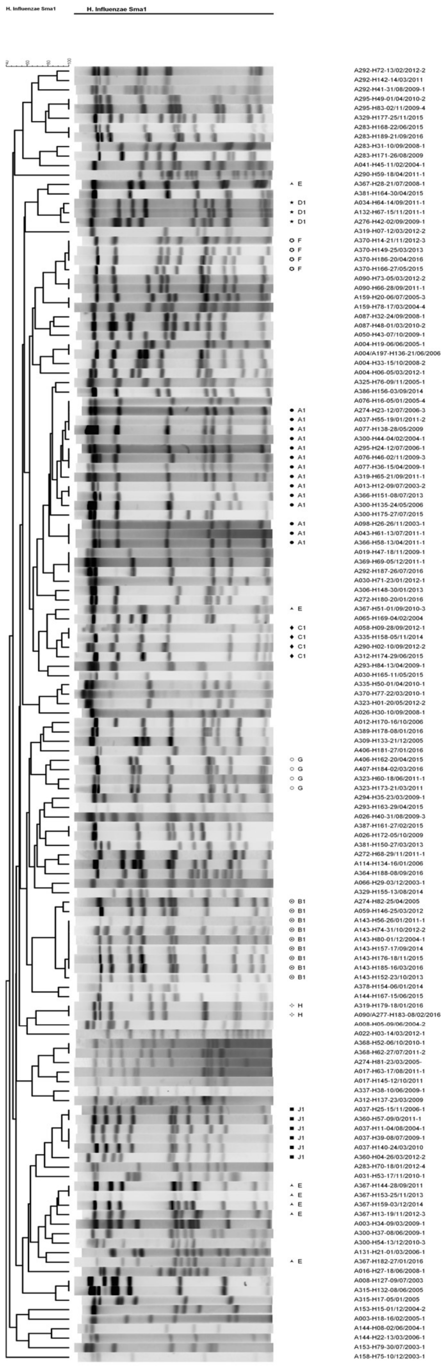

Supplementary Figure S1. Dendrogram showing pulsotype relationships among all typed isolates. Shared pulsotypes are indicated by letters and symbols. The dendrogram was generated using 2% tolerance with the UPGMA method. Isolate names are given in the format "Patient Number"-"Isolate Number"-"Collection Date (DD-MM-YYYY)".

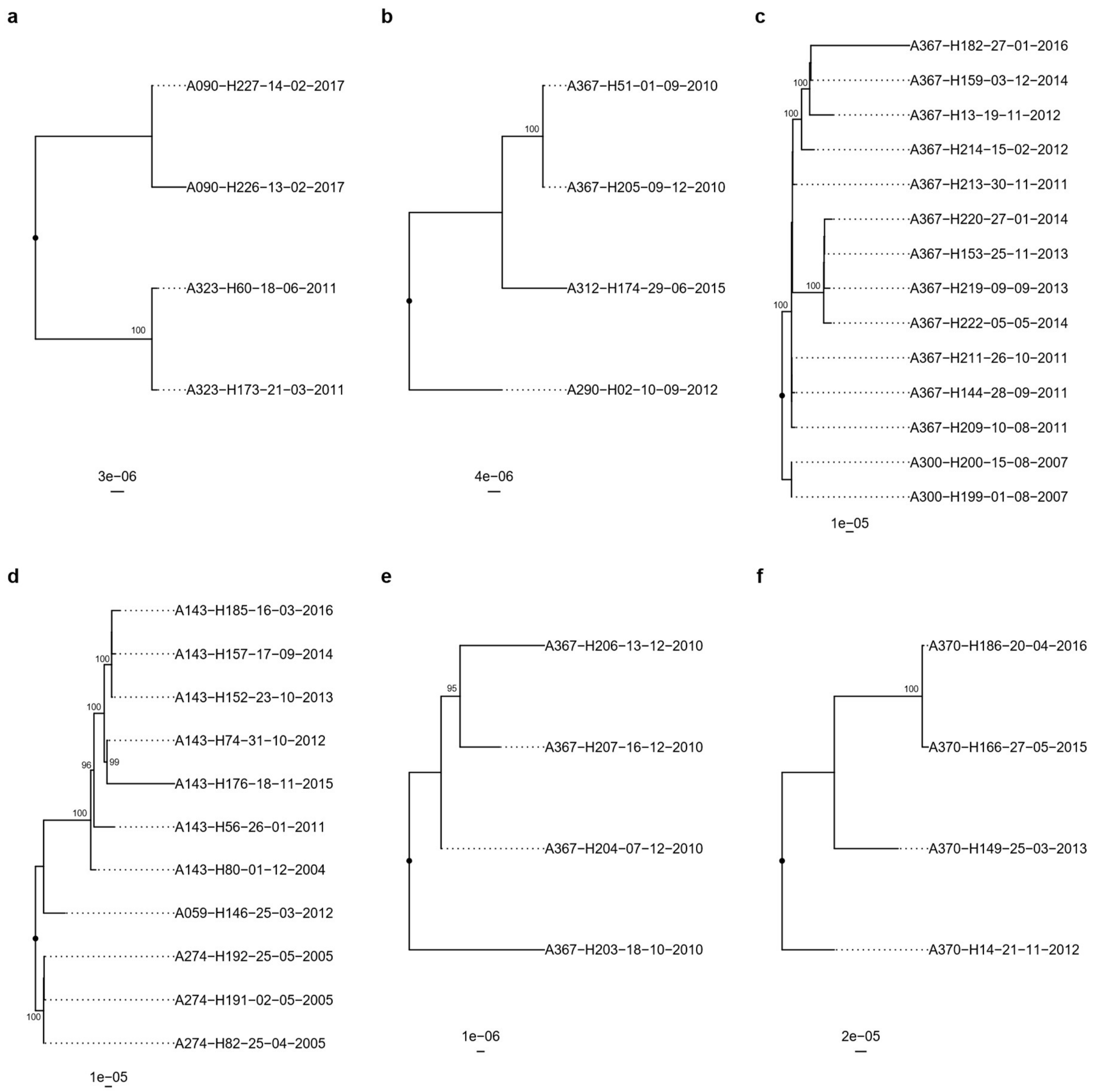

Supplementary Figure S2. Maximum likelihood consensus phylogenies for STs without patient-pairs of interest. (A) ST-11, (B) ST-14, (C) ST-105, (D) ST-124, (E) ST-203, and (F) ST-Novel 1. Isolate names are given in the format "Patient Number"-"Isolate Number"-"Collection Date (DD-MM-YYYY)". Scale bars for each tree are given in the units SNPs per site. Branch nodes with >95% Ultrafast bootstrap support are labelled.

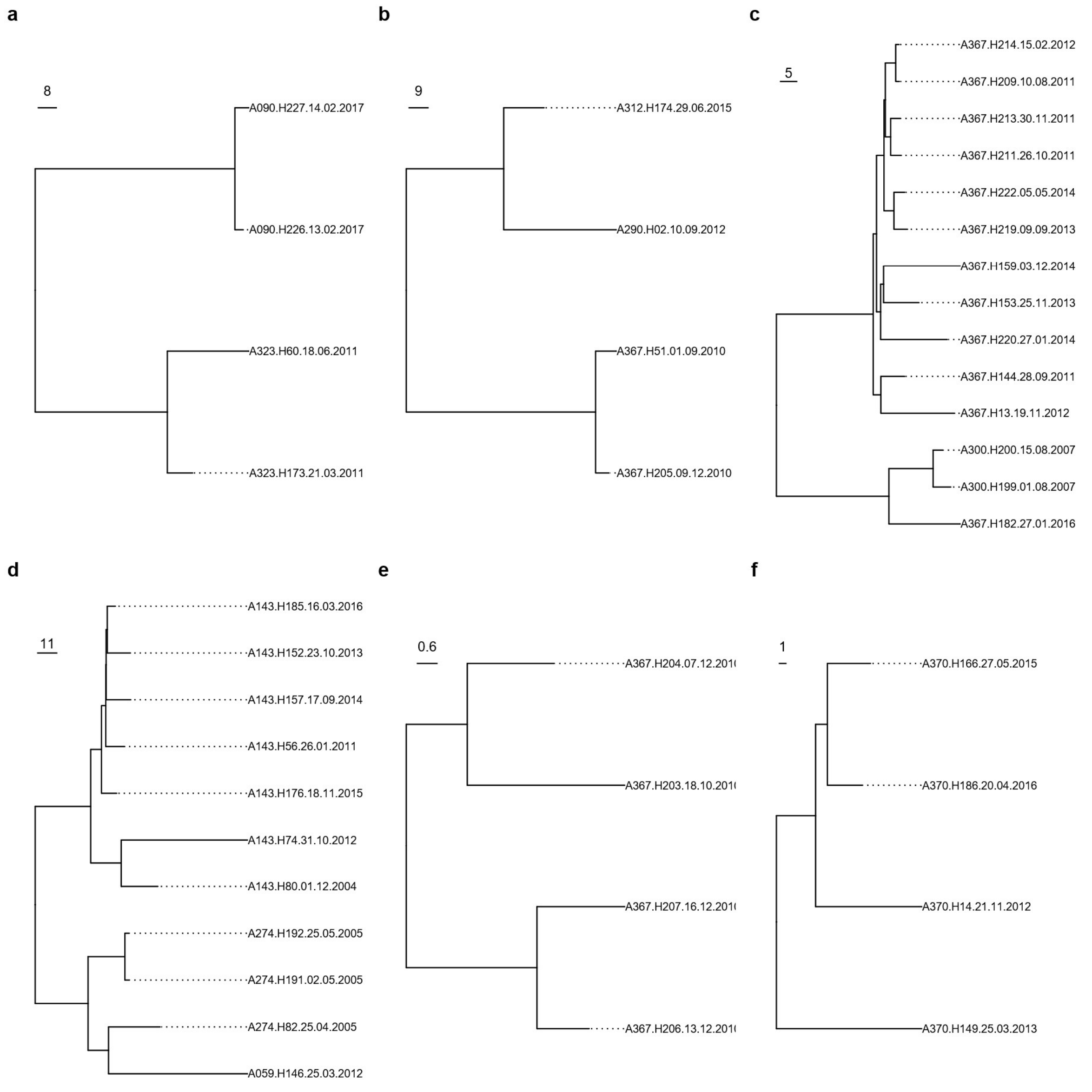

Supplementary Figure S3. Neighbor-joining trees for STs without patient-pairs of interest generated using gene presence/absence data. (A) ST-11, (B) ST-14, (C) ST-105, (D) ST-124, (E) ST-203, and (F) ST-Novel 1. Isolate names are given in the format "Patient Number"-"Isolate Number"-"Collection Date (DD-MM-YYYY)". Scale bars for each tree are given in number of differences in gene content.

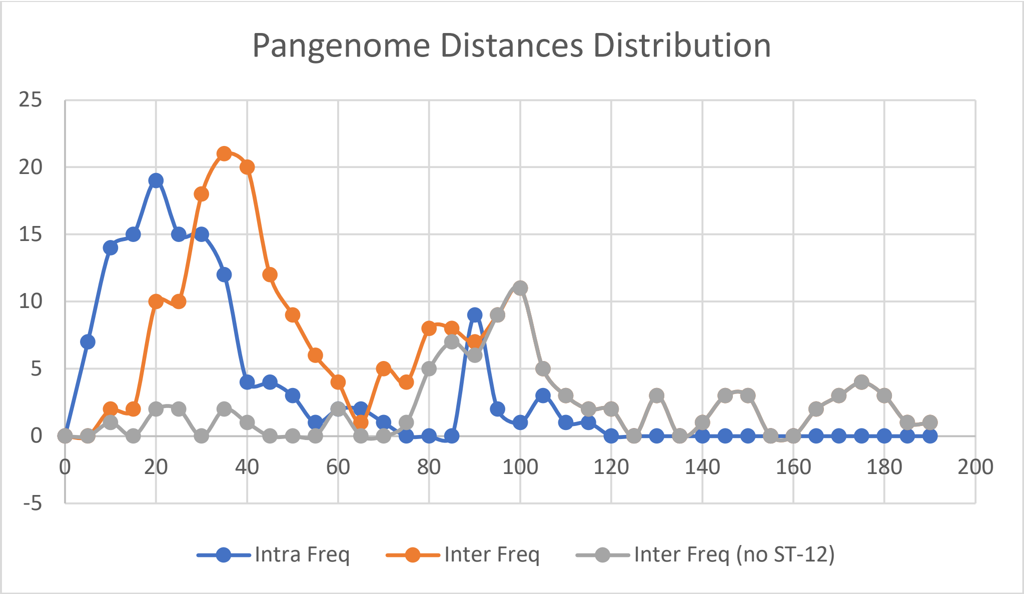

Supplementary Figure S4. Distributions of pairwise intra- and inter-patient differences in gene content. Intra-patient comparisons are shown in blue, inter-patient comparisons in orange, and inter-patient comparisons without ST-12 in grey.

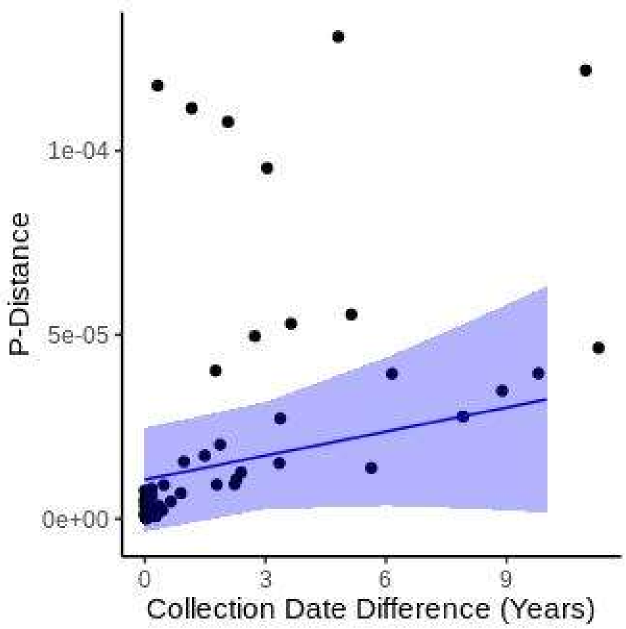

Supplementary Figure S5. Linear mixed model regression of p-distances and time between collection dates (years) for intra-ST isolate pairs. Data from all STs was included in the regression, except for p-distances of isolates suspected of being hypermutators (not plotted). Time between collection dates was fit as a fixed effect, and the intercept varied by patient (patient ID = random effect). Blue ribbon represents the 95% confidence interval.
