# Supplementary material for: The natural history and genetic diversity of Haemophilus influenzae infecting the airways of adults with cystic fibrosis
Source: Sci Rep. 2022 Sep 21;12:15765. doi: 10.1038/s41598-022-19240-2 (PMC9492733; doi:10.1038/s41598-022-19240-2)
Supplement: Supplementary file 2 — Supplementary Information 2. [file 41598_2022_19240_MOESM2_ESM.pdf]

Supplementary Methods

Pulsed-Field Gel Electrophoresis (PFGE) of *H. influenzae* Isolates

As a preliminary screen of genetic relatedness, first, last, and serial isolates (collected at yearly intervals between 2002-2016) from all pwCF with *H. influenzae* positive sputum cultures within the biobank were identified and subjected to PFGE using protocols adapted from Parkins et al.[1]. Viable isolates were grown from frozen cultures on chocolate agar with 5% CO2 overnight at 37°C. Isolate DNA was digested with 20U SmaI for 4 hours at 25°C. Samples were run on 1% SeaKem Gold agarose at 6V with a reorientation angle of 120° and initial and final switch times of 1 second and 20 seconds, respectively, for a total run time of 21 hours. PFGE gels were stained with GelRed (Biotium 41003), and banding patterns visualized using BioNumerics (v. 7.6) (Applied Maths, Belgium). Dendrograms were generated with 2% position tolerance and 1.5% optimization using the unweighted pair-group method with arithmetic mean and relatedness was quantified using the Sørensen-Dice similarity coefficient. Isolates with banding patterns with ≥80% similarity and ≤3 band differences were considered to represent the same strain[2] and were identified as “shared” if identified from ≥2 patients.

DNA Extraction and Whole-Genome Sequencing

Forty-two isolates belonging to shared pulsotypes, as well as 33 isolates not typed by PFGE but suspected of belonging to shared pulsotypes based on collection times, were selected for whole-genome sequencing (WGS). A further 11 isolates from two patients exhibiting prolonged carriage of the same pulsotype (A370 n=4, A367 n=7) were selected for WGS as controls to assess intra-patient diversity over time. Single colonies were grown as a lawn on chocolate agar from frozen cultures overnight at 37°C with 5% CO_2. Lawn scrapes were resuspended in 50 mM EDTA and centrifuged at 13000 rpm for 2 minutes. Genomic DNA extraction was then performed using the Promega Wizard® Genomic DNA Purification Kit according to the manufacturer’s instructions. Sequencing libraries were prepared using the Nextera XT DNA Library Prep Kit and sequenced on a MiSeq V3 Illumina sequencer (2x300 bp reads). A single isolate (A290-H059-18-04-2011) collected on suspicion of belonging to a shared pulsotype was subsequently found to be *H. haemolyticus* and excluded from all analyses. A second *H. influenzae* isolate (A058-H217-08-10-2012) produced a poor *de novo* assembly (>1000 contigs) and was also excluded from all analyses. Sequencing reads are available from NCBI (BioProject PRJNA770358).

Publicly Available Genomes

Isolates belonging to STs from patient-pairs of interest (see Determination of Relatedness below) were analyzed alongside public genomes as a measure of relative diversity of isolates from this study. Publicly available genomes with the same ST as those identified herein are presented in **Supplementary Table 1** and were obtained from the datasets of Cleary et al.[3], De Chiara et al.[4], Deghmane et al.[5], Moleres et al.[6], Pettigrew et al.[7] and Potts et al.[8]. Genomes from Cleary et al., Deghmane et al., and Potts et al. were downloaded from the European Nucleotide Archive (ENA)

as fastq files and processed as below. Genomes from De Chiara et al., Moleres et al., and Pettigrew et al. were downloaded from NCBI and sequencing reads simulated using ART_Illumina[9] (v. 2.5.8) with the following command line options: -l 250 -p -m 400 -s 110 -f 100 -rs 42. Simulated reads were then processed as below.

Read Quality Analysis and Trimming

Sequencing quality of raw reads from the 86 *H. influenzae* isolates sequenced in this study was analyzed using FastQC[10] (v. 0.11.9) and read trimming was subsequently performed using Trimmomatic[11] (v. 0.39) to remove sequencing adapters (option ILLUMINACLIP:/path/to/adapters/file:2:30:10:8:true), remove the 301st base (option CROP:300), trim reads from the 3' end if the average Phred quality fell below 5 in a sliding window of 4 bp (option SLIDINGWINDOW:4:5), and remove reads shorter than 30 bp after all previous trimming steps (option MINLEN:30).

***In silico* Multi-locus Sequence Typing (MLST)**

Isolates were typed by *in silico* MLST using their trimmed sequencing reads as input to stringMLST[12] (v. 0.6.3) against the *H. influenzae* MLST database downloaded using stringMLST.py --get-mlst. stringMLST.py --predict was then run with default settings to assign a sequence type to each isolate.

***De Novo* Assembly and Genome Annotation**

Sequencing depth was estimated from forward reads from each isolate by dividing the total number of bases in the forward reads fastq file by the estimated genome size from Mash[13] (v. 1.1). Mash was run with the following options: -k 32 -m 3 -s 1000.

De novo assembly of isolate genomes was performed with Unicycler[14] (v. 0.4.8). Unicycler was run with default settings in normal mode (option --mode normal), except the --depth-filter parameter was set to 0.01 to remove only contigs with a sequencing depth below 1% of the chromosomal depth.

Assembled genomes of isolates from this study were polished using NextPolish[15] (v. 1.3.0) using a custom read alignment step, in which isolate reads were first aligned to the assembled genome using BWA[16] (v. 0.7.17-r1188) and Samtools[17] (v. 1.8). NextPolish was run in two iterations of algorithmic steps 1 and 2, which were run successively in each iteration. Additionally, ploidy (option --ploidy) was set to 1. Assembled public genomes were not polished.

All assembled genomes were annotated using RASTtk as implemented in the PATRIC Command Line Interface[18] (v1.035) using the default annotation workflow with the addition of prophage calling using PhiSpy.

Pangenome Analysis

Panaroo[19] (v. 1.2.7) was used to analyze gene content differences between isolates as well as categorize orthologs into the core and accessory genomes. Genome annotations in GFF3 format were used as input to Panaroo, which was run with the following options: -a pan, --aligner mafft, --core_threshold 1.0, --threshold 0.98, --clean-mode moderate, and --remove-invalid-genes. Panaroo output was then subset by ST and clustering of gene presence/absence data was performed in R (v. 3.6.3) using pairwise Manhattan distances and the Neighbor-Joining algorithm as implemented in the Ape package[20] (v. 5.3).

SNP and Phylogenetic Analysis

Single-nucleotide polymorphism (SNP) calling was performed using Snippy[21] (v. 4.6.0) in an ST-specific manner with annotated same-ST draft assemblies used as references for all STs except ST-321, for which only two isolates were available and a complete same-ST reference genome was used (RefSeq Accession GCF_000968335.1) (**Supplementary Table 2**). Pseudo-whole genome alignments generated by snippy-core were used as input to IQ-Tree[22] (v. 2.0.3), which was used to generate maximum likelihood phylogenies for each ST. IQ-Tree was run with 10000 UltraFast bootstrap replicates[23], and the best-fitting model of nucleotide substitution (as identified by the Bayesian Information Criterion) was selected using the IQ-Tree ModelFinder[24]. Recombination was subsequently identified, and recombinant regions masked, using ClonalFrameML[25] (v. 1.12) and the maskrc-svg[26] (v. 0.5) python script, respectively. Maximum likelihood phylogenies, along with their pseudo-whole genome alignments, were used as input to ClonalFrameML, which was run with default settings. Maskrc-svg was then used to create recombination-masked pseudo-whole genome alignments, from which pairwise SNP distances were calculated using snp-dists[27] (v. 0.7.0) and pairwise p-distances were calculated using MEGA X[28] (v. 10.2.4). IQ-Tree was then used to generate new bootstrap consensus phylogenies using the recombination-masked alignments as above.

Identification of Putative Hypermutators

Putative hypermutating isolates were identified by analysis of transition (Ts)/transversion (Tv) mutational ratios and mutations in hypermutation associated genes on a per-ST basis. Ts/Tv ratios were estimated using VCFTools[29] (v. 0.1.6) using VCF files generated using SNP-sites[30] (v. 2.5.1) from ST-specific recombination masked alignments generated above. Mutations in the following genes associated with hypermutation were identified using custom Python scripts: DNA polymerase III epsilon subunit (*dnaQ*), DNA mismatch repair system genes (DNA adenine methyltransferase *dam*, *mutH*, *mutL*, *mutS*, DNA helicase II *uvrD*), GO system genes (*mutM*, *mutT*, *mutY*), and DNA polymerase I (*polA*)[31]. Isolates with elevated Ts/Tv ratios were deemed potential hypermutators[32].

Determination of relatedness for inference of possible transmission details

For the first tier, a p-distance threshold was calculated as a preliminary measure to rule out the possibility of transmission between isolate pairs (after removing outliers/possible hypermutators) using the approach of Coll et al.[33]. In brief, the threshold was calculated by fitting a linear mixed effects model to the data, based on time between isolate collection dates and pairwise p-distances. Pairwise p-distance between isolates was treated as the dependent variable, the time between collection dates as the independent variable (fixed effect), and the intercept was allowed to vary by patient (random effect). The intercept of the model was interpreted as quantifying intra-patient diversity, and the slope represented the evolutionary rate (rate of substitution accumulation). A final threshold was calculated as the 95th percentile of the intercept plus the number of mutations expected to accumulate over an equivalent period of 18 months, conditional on the fact that isolates of the pair must have been collected within 6 months of each other. This corresponds to a scenario where on one extreme, if two isolates are collected 6 months apart, then the earlier isolate was collected no more than 6 months from the pair's tMRCA and the later no more than 1 year. On the other extreme, if both isolates are collected on the same day, then each may have been collected up to 9 months from the pair's tMRCA.

As pwCF are routinely followed at quarterly intervals at our clinic, we reasoned that fewer than 6 months should separate the isolates of any two patients involved in a transmission event, and that neither infection in the transmitting or receiving patient would go undetected (ie culture negative) for more than 6 months. We further allowed for a 6-month gap between collection dates so as to account for the time between subsequent patient visits. Thus, the final calculation was: 95th percentile of intercept plus 1.5 times the number of mutations expected to accumulate over 1 year, or 18 months worth of evolution. The linear mixed effects model was fitted using the lme4[34] R package (v. 1.1-26). Visualization of the regression was performed using the Effects[35] (v. 4.2-0) and ggplot2[36] (v. 3.2.1) R packages.

1. Parkins, M. D. *et al.* Twenty-Five-Year Outbreak of Pseudomonas aeruginosa Infecting Individuals with Cystic Fibrosis: Identification of the Prairie Epidemic Strain. *J. Clin. Microbiol.* **52**, 1127–1135 (2014).
2. Tenover, F. C. *et al.* Interpreting chromosomal DNA restriction patterns produced by pulsed-field gel electrophoresis: criteria for bacterial strain typing. *J. Clin. Microbiol.* **33**, 2233–2239 (1995).
3. Cleary, D. *et al.* Pneumococcal vaccine impacts on the population genomics of non-typeable Haemophilus influenzae. *Microb. Genomics* **4**, e000209 (2018).
4. De Chiara, M. *et al.* Genome sequencing of disease and carriage isolates of nontypeable Haemophilus influenzae identifies discrete population structure. *Proc. Natl. Acad. Sci. U. S. A.* **111**, 5439–5444 (2014).
5. Deghmane, A.-E. *et al.* High diversity of invasive Haemophilus influenzae isolates in France and the emergence of resistance to third generation cephalosporins by alteration of ftsI gene. *J. Infect.* **79**, 7–14 (2019).
6. Moleres, J. *et al.* Antagonistic Pleiotropy in the Bifunctional Surface Protein FadL (OmpP1) during Adaptation of Haemophilus influenzae to Chronic Lung Infection Associated with Chronic Obstructive Pulmonary Disease. *mBio* **9**, e01176-18 (2018).
7. Pettigrew, M. M. *et al.* Haemophilus influenzae genome evolution during persistence in the human airways in chronic obstructive pulmonary disease. *Proc. Natl. Acad. Sci.* **115**, E3256–E3265 (2018).
8. Potts, C. C. *et al.* Genomic characterization of Haemophilus influenzae: a focus on the capsule locus. *BMC Genomics* **20**, 733 (2019).

9. Huang, W., Li, L., Myers, J. R. & Marth, G. T. ART: a next-generation sequencing read simulator. *Bioinformatics* **28**, 593–594 (2012).

10. *FastQC*. (Babraham Bioinformatics).

11. Bolger, A. M., Lohse, M. & Usadel, B. Trimmomatic: a flexible trimmer for Illumina sequence data. *Bioinformatics* **30**, 2114–2120 (2014).

12. Gupta, A., Jordan, I. K. & Rishishwar, L. stringMLST: a fast k-mer based tool for multilocus sequence typing. *Bioinformatics* **33**, 119–121 (2017).

13. Ondov, B. D. *et al.* Mash: fast genome and metagenome distance estimation using MinHash. *Genome Biol.* **17**, 132 (2016).

14. Wick, R. R., Judd, L. M., Gorrie, C. L. & Holt, K. E. Unicycler: Resolving bacterial genome assemblies from short and long sequencing reads. *PLOS Comput. Biol.* **13**, e1005595 (2017).

15. Hu, J., Fan, J., Sun, Z. & Liu, S. NextPolish: a fast and efficient genome polishing tool for long-read assembly. *Bioinformatics* **36**, 2253–2255 (2020).

16. Li, H. & Durbin, R. Fast and accurate short read alignment with Burrows-Wheeler transform. *Bioinforma. Oxf. Engl.* **25**, 1754–1760 (2009).

17. Li, H. *et al.* The Sequence Alignment/Map format and SAMtools. *Bioinformatics* **25**, 2078–2079 (2009).

18. Davis, J. J. *et al.* The PATRIC Bioinformatics Resource Center: expanding data and analysis capabilities. *Nucleic Acids Res.* **48**, D606–D612 (2020).

19. Tonkin-Hill, G. *et al.* Producing polished prokaryotic pangenomes with the Panaroo pipeline. *Genome Biol.* **21**, 180 (2020).

20. Paradis, E., Claude, J. & Strimmer, K. APE: Analyses of Phylogenetics and Evolution in R language. *Bioinformatics* **20**, 289–290 (2004).

21. Seemann, T. *Snippy*.

22. Nguyen, L.-T., Schmidt, H. A., von Haeseler, A. & Minh, B. Q. IQ-TREE: A Fast and Effective Stochastic Algorithm for Estimating Maximum-Likelihood Phylogenies. *Mol. Biol. Evol.* **32**, 268–274 (2015).

23. Hoang, D. T., Chernomor, O., von Haeseler, A., Minh, B. Q. & Vinh, L. S. UFBoot2: Improving the Ultrafast Bootstrap Approximation. *Mol. Biol. Evol.* **35**, 518–522 (2018).

24. Kalyaanamoorthy, S., Minh, B. Q., Wong, T. K., von Haeseler, A. & Jermiin, L. S. ModelFinder: Fast Model Selection for Accurate Phylogenetic Estimates. *Nat. Methods* **14**, 587–589 (2017).

25. Didelot, X. & Wilson, D. J. ClonalFrameML: Efficient Inference of Recombination in Whole Bacterial Genomes. *PLoS Comput. Biol.* **11**, (2015).

26. Kwong, J. & Seemann, T. *maskrc-svg*.

27. Seemann, T. *snp-dists*.

28. Kumar, S., Stecher, G., Li, M., Knyaz, C. & Tamura, K. MEGA X: Molecular Evolutionary Genetics Analysis across Computing Platforms. *Mol. Biol. Evol.* **35**, 1547–1549 (2018).

29. Danecek, P. *et al.* The variant call format and VCFtools. *Bioinformatics* **27**, 2156–2158 (2011).

30. Page, A. J. *et al.* SNP-sites: rapid efficient extraction of SNPs from multi-FASTA alignments. *Microb. Genomics* **2**, e000056 (2016).

31. Oliver, A. & Mena, A. Bacterial hypermutation in cystic fibrosis, not only for antibiotic resistance. *Clin. Microbiol. Infect.* **16**, 798–808 (2010).

32. Veschetti, L., Sandri, A., Krogh Johansen, H., Lleò, M. M. & Malerba, G. Hypermutation as an Evolutionary Mechanism for Achromobacter xylosoxidans in Cystic Fibrosis Lung Infection. *Pathogens* **9**, 72 (2020).

33. Coll, F. *et al.* Definition of a genetic relatedness cutoff to exclude recent transmission of meticillin-resistant Staphylococcus aureus: a genomic epidemiology analysis. *Lancet Microbe* **1**, e328–e335 (2020).

34. Bates, D., Mächler, M., Bolker, B. & Walker, S. Fitting Linear Mixed-Effects Models Using lme4. *J. Stat. Softw.* **67**, 1–48 (2015).

35. Weisberg, F. J. *An R Companion to Applied Regression*. (Sage, 2019).

36. Wickham, H. *ggplot2: Elegant Graphics for Data Analysis*. (Springer-Verlag New York, 2016).
